# Supplementary figures and images for: Autophagy and Programmed Cell Death Modalities Interplay in HIV Pathogenesis
Source: Cells. 2025 Feb 28;14(5):351. doi: 10.3390/cells14050351 (PMC11899401; doi:10.3390/cells14050351)

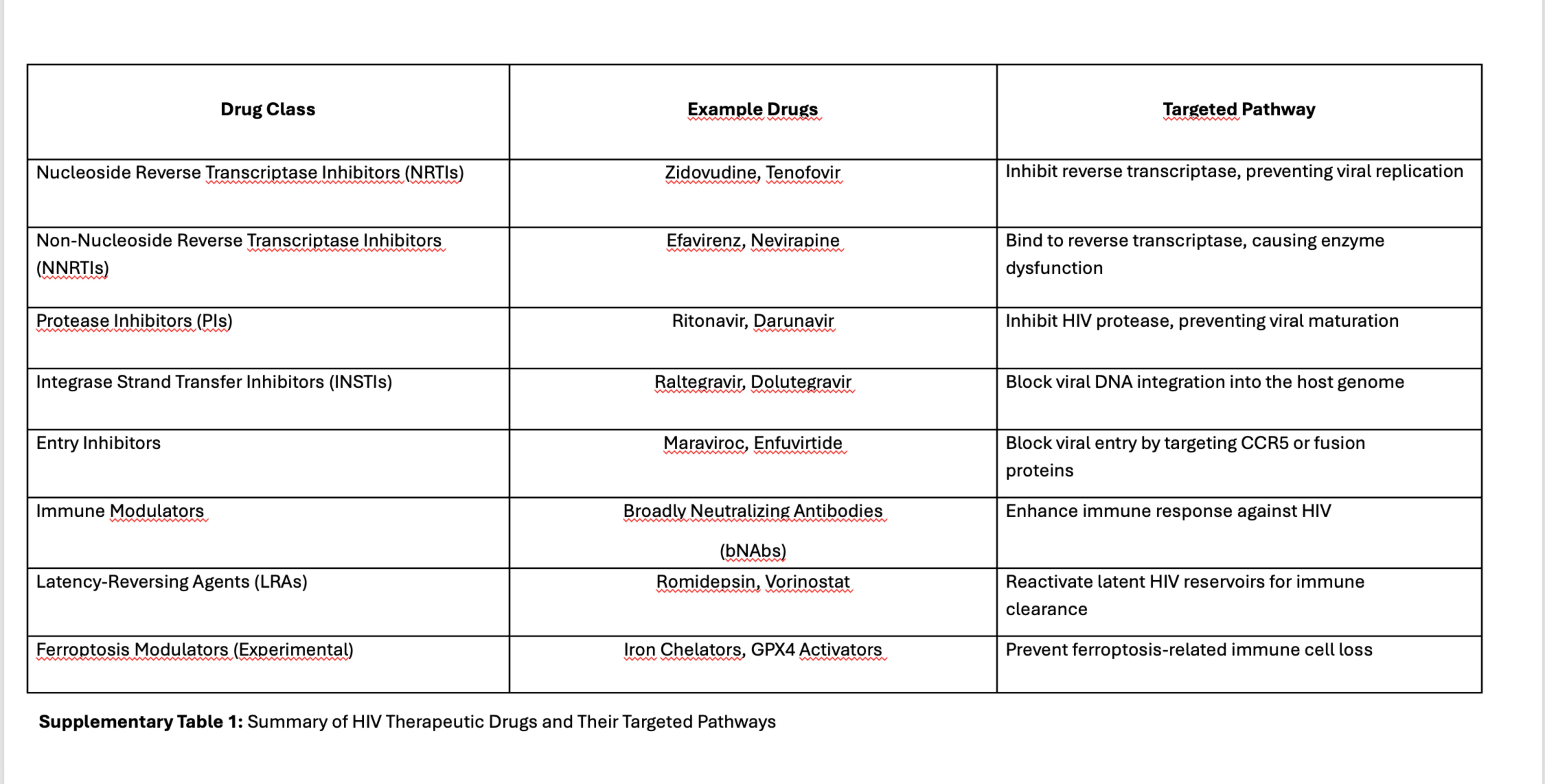

Supplement: Supplementary file 1 [file cells-14-00351-s001.zip › Table S1.jpg]
